# The Kindlin-2 regulation of epithelial-to-mesenchymal transition in breast cancer metastasis is mediated through miR-200b

**DOI:** 10.1038/s41598-018-25373-0

**Published:** 2018-05-09

**Authors:** Khalid Sossey-Alaoui, Elzbieta Pluskota, Dorota Szpak, William P. Schiemann, Edward F. Plow

**Affiliations:** 1 Department of Molecular Cardiology, Lerner Research Institute, Cleveland Clinic, Cleveland, OH USA; 2 Case Comprehensive Cancer Center, Cleveland, OH USA

## Abstract

Metastasis is the main cause of death in cancer patients, including breast cancer (BC). Despite recent progress in understanding the biological and molecular determinants of BC metastasis, effective therapeutic treatments are yet to be developed. Among the multitude of molecular mechanisms that regulate cancer metastasis, the epithelial-to-mesenchymal transition (EMT) program plays a key role in the activation of the biological steps leading to the metastatic phenotype. Kindlin-2 has been associated with the pathogenesis of several types of cancers, including BC. The role of Kindlin-2 in the regulation of BC metastasis, and to a lesser extent in EMT is not well understood. In this study, we show that Kindlin-2 is closely associated with the development of the metastatic phenotype in BC. We report that knockout of Kindlin-2 in either human or mouse BC cells, significantly inhibits metastasis in both human and mouse models of BC metastasis. We also report that the Kindlin-2-mediated inhibition of metastasis is the result of inhibition of expression of key molecular markers of the EMT program. Mechanistically, we show that miR-200b, a master regulator of EMT, directly targets and inhibits the expression of Kindlin-2, leading to the subsequent inhibition of EMT and metastasis. Together, our data support the targeting of Kindlin-2 as a therapeutic strategy against BC metastasis.

## Introduction

Metastasis is the leading cause for cancer-related death worldwide, including breast cancer (BC)-related mortalities^1^. Metastatic BC accounts for ~41,000 deaths and more than 250,000 new cases of invasive BC reported each year in the United States^1^. For patients with metastatic BC, the median survival is only 1.5 to 3 years. Several biological factors are involved that lead to cancer metastasis, including the epithelial-mesenchymal transition (EMT) program^2^. EMT is a normal biological process during embryonic development and wound healing^3^. However, in pathological conditions, including the process of tumorigenesis, EMT, by activating cancer cell invasion, cancer stem cells, and drug resistance contributes to malignancy^3–8^. EMT also plays a critical role in cancer metastasis by allowing cancer cells to colonize distant organs and establish secondary tumours^9^.

Kindlin-2 is an adaptor protein that belong to the family of 4.1-ezrin-ridixin-moesin (FERM) domain containing proteins (reviewed in^10^). Recent studies have implicated Kindlin-2 in the pathology of several cancers, including the one originating from the breast (reviewed in^11,12^). Our recent study showed that Kindlin-2 is involved in the growth of primary BC tumours by regulating the interaction between the tumour and tumour microenvironment^13^. The role of Kindlin-2 in EMT and BC metastasis, however, is not well understood. In the present study, we show that Kindlin-2 is also involved in the process of BC metastatic colonization to the lung by regulating the EMT program downstream of miR-200b. We previously reported that expression levels of Kindlin-2 are increased in the more aggressive, highly metastatic human and murine breast cancer (BC) cell lines and in human and mouse BC progression series. Kindlin-2 expression was also elevated in BC biopsy tissues, and its levels correlated with reduced survival in human BC patients^13^. We also showed that CRISPR/Cas9-mediated Kindlin-2 knockout in human MDA-MB-231 and murine 4T1 cell lines inhibited their invasive and migratory properties *in vitro* and tumour growth *in vivo*^13^. Here, we report that loss of expression of Kindlin-2 inhibited metastasis in both spontaneous and experimental metastasis BC models. Suppression of metastasis correlated with inhibition of EMT and downregulation of EMT genes; e.g. Fibronectin, N-Cadherin and Vimentin, in both human and mouse BC metastasis models. Mechanistically, we found the Kindlin-2 regulation of EMT is mediated through microRNA miR-200b, an established master regulator of EMT^9,14^. miR-200b specifically targets and regulates Kindlin-2 expression through a conserved seed sequence in the 3′UTR in both the human and mouse FERM2 genes, and expression levels of miR-200b inversely correlate with that of Kindlin-2 and with the aggressiveness of BC cell lines. Importantly, overexpression of miR-200b in MDA-MB-231 and 4T1 cells inhibited Kindlin-2 expression, concomitant with downregulation of EMT genes, while it also resulted in a significant reduction of the metastatic burden *in vivo*.

Collectively, our data provide the underpinnings of a new signalling axis, in which miR-200b regulates Kindlin-2, which in turn regulates EMT, thereby suggesting a novel role of Kindlin-2 in the regulation of BC progression and metastasis.

## Results

### Kindlin-2 is required for cancer cell adhesion, spreading and focal adhesion formation

**Figure 1:**
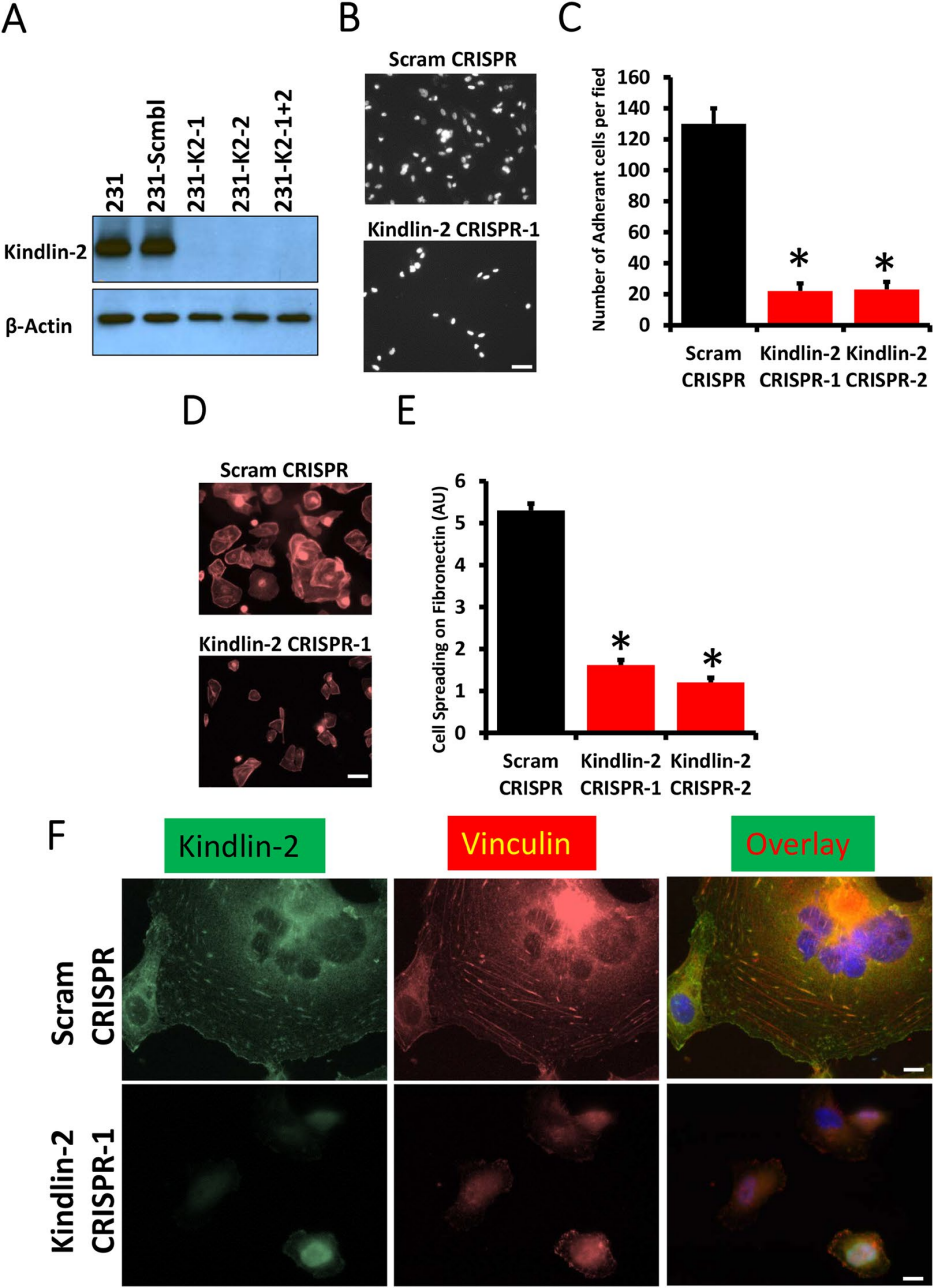
Kindlin-2 is required for cancer cell adhesion, spreading and focal adhesion formation. (**A**) Western blots developed with anti-Kindlin-2 antibody of protein lysates from MDA-MB-231 transduced with a scrambled sgRNA (Scram CRISPR), Kindlin-2 sgRNA-1 (K2 CRISPR-1), Kindlin-2 sgRNA-2- (K2 CRISPR-2) or both sgRNA-1 and -2 (K2-CRISPR-1 + 2). β-Actin is a loading control. (**B**,**C**) Cell adhesion assay: Control (Scram CRISPR) or Kindlin-2-deficient (Kindlin-2 CRISPR-1 and -2) MDA-MB-21 cells were seeded onto on fibronectin-coated slides for 1 h, at which point they were fixed and stained with phalloidin-568 to visualize actin filaments. Micrographs of K2-CRISPR-1 are shown as an example in A and quantification is shown in B. (**D**,**E**) Cell spreading assay: Control (Scram CRISPR) or Kindlin-2-deficient (Kindlin-2 CRISPR-1 and -2) MDA-MB-21 cells were seeded onto on fibronectin-coated slides for 2 h, at which point they were fixed and stained with phalloidin-568 to visualize actin filaments. Micrographs of K2-CRISPR-1 are shown as an example in **D** and quantification is shown in **E**. (**F**) Representative confocal microscopy micrographs of Control (Scram CRISPR, top panels) or Kindlin-2-deficient (Kindlin-2 CRISPR-1, bottom panels) MDA-MB-21 cells that were stained for Kindin-2 (left panels) and Vinculin (middle panels). Co-localization of both Kindlin-2 and Vinculin at the focal adhesion structures is shown as yellow puncta in the overlay right panels. Data are the means ± SEM, N = 3; *p < 0.05; Student’s t-test).

Several cell functions are required for cancer cell invasion and metastasis, including adhesion and spreading. Kindlin-2, like the other members of the Kindlin family was shown to regulate cell spreading and adhesion, downstream of integrins in different cell types. This function has, however, not been established in breast cancer cells. Our recently published study has shown that Kindlin-2 is overexpressed in aggressive human and mouse breast cancer cells and tumours, and that Kindlin-2 is required for primary tumour growth in both human and murine BC models^13^. The main goal of this study was to investigate whether the function of Kindlin-2 in BC tumour progression and metastasis is linked to the activation of cell spreading and adhesion. We used CRISPR/Cas9 gene editing to generate several clones lacking Kindlin-2 expression in MDA-MB-231 cells (Fig. 1A and^13^). Loss of Kindlin-2 had no evident effect on cell proliferation^13^. When Kindlin-2-deficient (Kindlin-2 CRISPR-1 or-2) or the control (Scram CRISPR) MDA-MB-231 cells were seeded on fibronectin-coated slides, the number of Kindlin-2-deficient cells that adhered was more that 6-fold lower (p < 0.01) than that of the control cells (Fig. 1B,C). Cell spreading was also significantly affected by loss of Kindlin-2; the average surface of the Kindlin-2 CRISPR MDA-MB-231 cells was more than 5-fold smaller (p < 0.01) than that of the control (Scram CRISPR) counterparts (Fig. 1D,E). Since focal adhesions are known to be required for cell adhesion and spreading, we used immuno-fluorescence staining of vinculin as a way to visualize focal adhesions in control or Kindlin-2-deficient MDA-MB-231 cells that were allowed to adhere and spread on fibronectin. Cells lacking kindlin-2 (Fig. 1F, lower panels) were unable to spread when compared to the control cells (Fig. 1F, upper panels), which confirm the results shown in Fig. 1D. We also found that in the control (Scram CRISPR) cells, Kindlin-2 to stain in a dot-like pattern (left panels), which nicely co-localized with the Vinculin structures (middle) in the merged image (Fig. 1F, right panels). These Vinculin structures that represent focal adhesions were abundant and evenly distributed throughout the surface of fully spread control cells, but were hardly detected in Kindlin-2-deficient cells (Fig. 1F, lower panels). Together, these data show that Kindlin-2 is required for cell spreading and adhesion of BC cells.

### Loss of Kindlin-2 inhibits invadopodia formation and degradation of extracellular matrix

**Figure 2:**
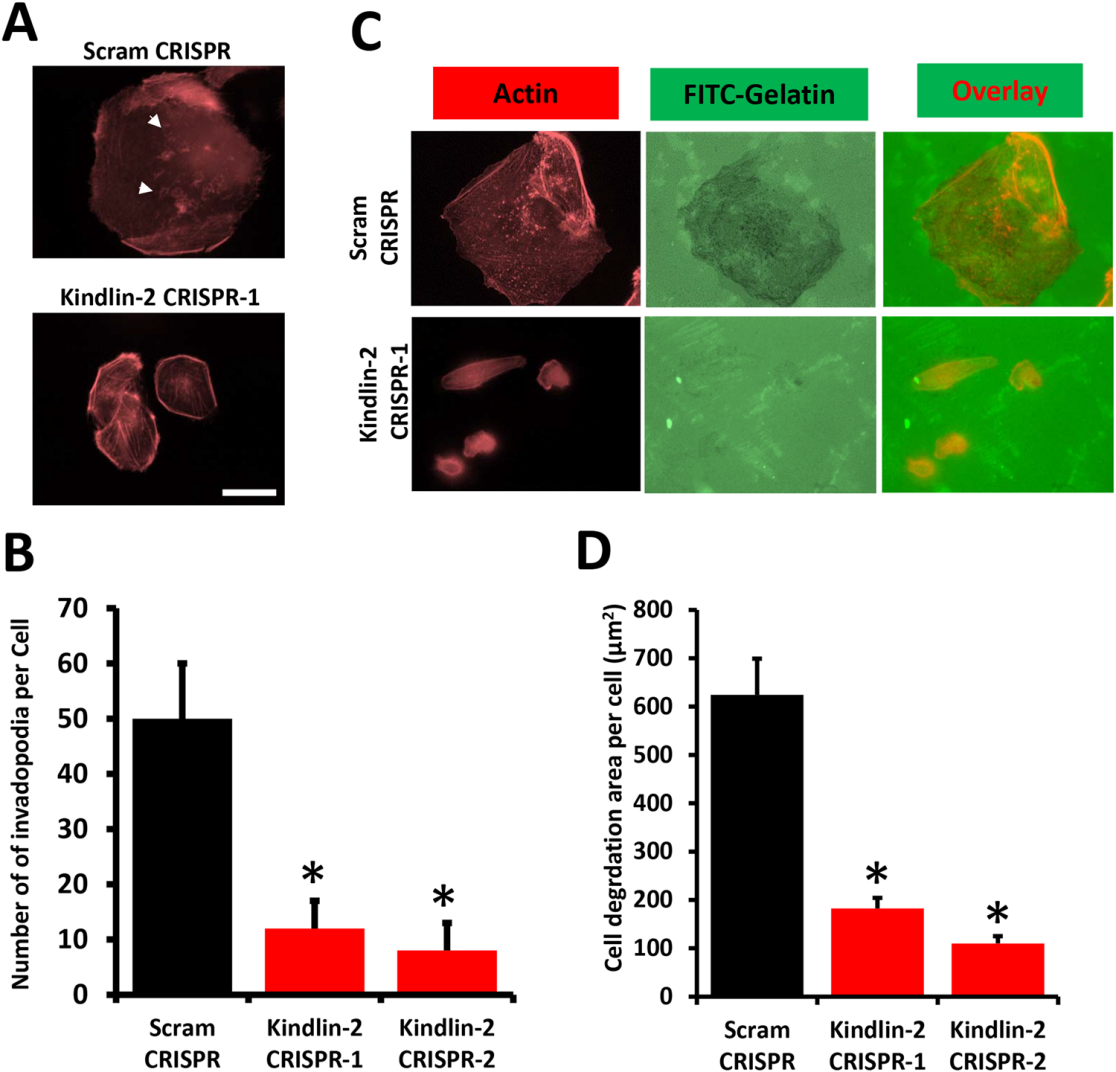
Loss of Kindlin-2 inhibits invadopodia formation and degradation of extracellular matrix. (**A**,**B**) Invadopodia formation assay: Control (Scram) or Kindlin-2-deficient (Kindlin-2 CRISPR-1 and -2) MDA-MB-21 cells were seeded onto FITC-conjugated Gelatine for 18 h, at which point they were fixed and stained with phalloidin-568 to visualize actin filaments. Micrographs of Kindlin-2 CRISPR-1 are shown as an example (**A**). Invadopodia structures shown as white dots (arrows) and rosettes (arrow-heads) were quantified (**B**). (**C**,**D**) Extracellular matrix degradation (ECM) assay: Cells were treated as in A. Invadopodia structures shown as white dots (C, left panels) and ECM degradation, shown as dark spots (C, middle panels), which coincided with invadopodia structures (C, right panels) and were quantified (**D**). Data are the means ± SEM, N = 3; *p < 0.05; Student’s t-test).

Our recently published study showed that Kindlin-2 is required for BC motility, migration and invasion, without affecting cell proliferation^13^. The biological significance of loss of Kindlin-2 was further investigated by assessing the ability of BC cells to form invadopodia and degrade the ECM. When control (Scram CRISPR) MDA-MB-231 cells were seeded on gelatine-coated coverslips, F-actin staining, revealed invadopodia as dot-like structures (Arrow heads) or rosettes (Arrows) on the surface of the cells (Fig. 2A, upper panel). The Kindlin-2-deficient (Kindlin-2 CRISPR-1) cells, on the other hand, formed fewer invadopodia structures (Fig. 2A, lower panel). In fact, loss of Kindlin-2 resulted in more than 5-fold reduction (p < 0.01) in the number of invadopodia (Fig. 2B). In cancer cells and other cell types, invadopodia coincide with sites of ECM degradation. Accordingly, we utilized this gelatine-based invadopodia assay to assess the effect of Kindlin-2 on the degradation of the ECM by MDA-MB-231 cells. Control or Kindlin-2-deficient MDA-MB-231 were seeded onto fluorescent (FITC) gelatine slides. Similar to data presented in Fig. 2A, staining for F-Actin showed that the control (Scram CRISPR) cells formed significantly higher (p < 0.01) number of invadopodia than the Kindlin-2-deficient (Kindlin-2 CRISPR-1) cells (Fig. 2C, left panel and Fig. 2D). These invadopodia structures overlapped with the sites of gelatine degradation, shown as dark areas where gelatine was depleted (Fig. 2C, middle panel). Loss of Kindlin-2 also resulted in a significant inhibition (p < 0.01) of ECM degradation (Fig. 2D). Expression and activity of metalloproteases (MMPs) are known to be required for EMC degradation at the invadopodia sites. We found loss of Kindlin-2 to also significantly inhibit the expression of MMP2 and MMP9 (Fig. S1 in Supplementary information). These results demonstrate the role of Kindlin-2 in the regulation of invadopodia formation and the invadopodia-mediated degradation of the ECM that is required for cancer cell motility and invasion.

### Kindlin-2 is required of BC metastasis in both the spontaneous and the experimental metastasis assays

**Figure 3:**
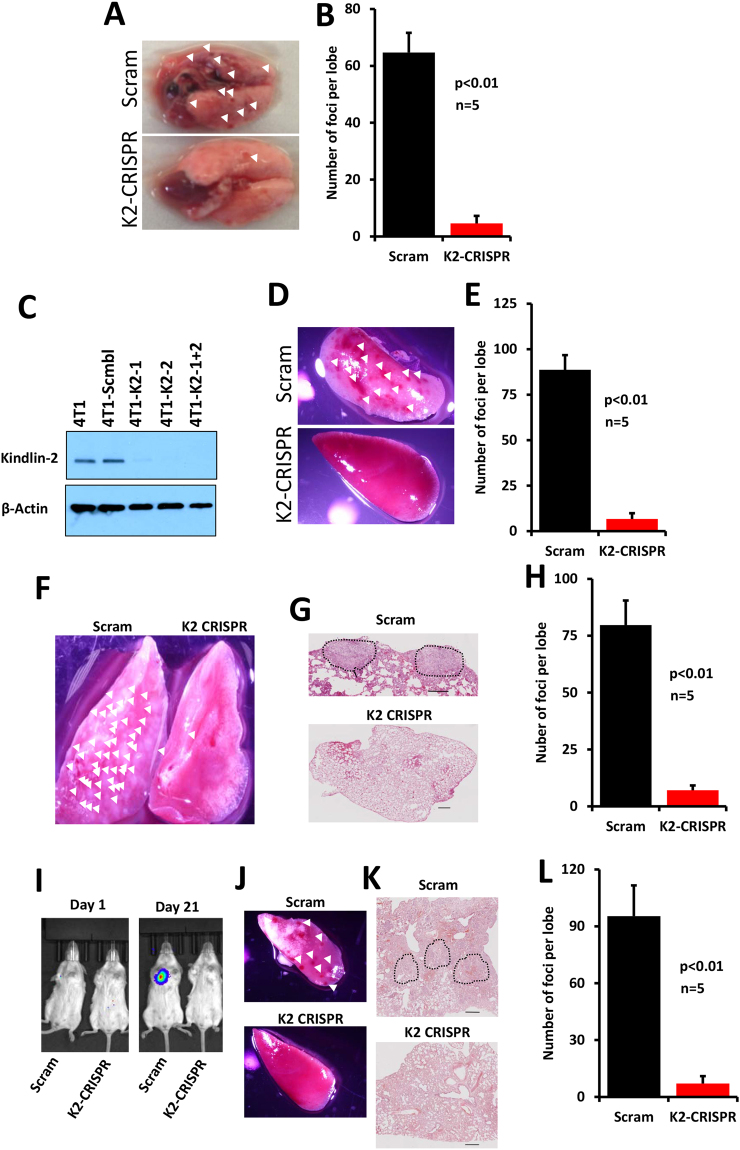
Loss of Kindlin-2 in BC cells inhibits metastasis to the lungs in both the spontaneous and the experimental metastasis assays. (**A**–**E**) The spontaneous metastasis assay: (**A**) Representative lungs of NSG mice that were implanted in the mammary fat pads with either control (Scram) or Kindlin-2-deficient (K2-CRISPR) MDA-MB-231 cells. Arrowheads point to metastasis foci which were quantified in **B**. (**C**) Western blots developed with anti-Kindlin-2 antibody of protein lysates from 4T1 cells transduced with a scrambled sgRNA (Scram CRISPR), Kindlin-2 sgRNA-1 (K2 CRISPR-1), Kindlin-2 sgRNA-2- (K2-CRISPR-2) or both sgRNA-1 and -2 (K2-CRISPR-1 + 2). β-Actin is a loading control. (**D**) Representative lungs of BALB/C mice that were implanted in the mammary fat pads with either control (Scram) or Kindlin-2-deficient (K2-CRISPR) 4T1 cells. Arrowheads point to metastasis foci which were quantified in **E**. (**F**–**L**) The lung colonization assay. (**F**) Representative lungs of NSG mice that were injected via tail vein with either control (Scram) or Kindlin-2-deficient (K2-CRISPR) MDA-MB-231 cells. Arrowheads point to metastasis foci. (**G**) H&E staining of sections of lungs shown in **F** where the metastasis foci are delineated by a dark broken line. (**H**) Quantification of the metastasis foci. (**I**) Luciferase bioluminescence of lung metastases of BALB/C mice that were injected via tail vein with either control (Scram) or Kindlin-2-deficient (K2-CRISPR) 4T1 cells. (**J**) Representative lungs from mice shown in I. Arrowheads point to metastasis foci. (**K**) H&E staining of sections of lung shown in J where the metastasis foci are delineated by a dark broken line. (**L**) Quantification of the metastasis foci. Data are the means ± SEM, n = 5 mice; *p < 0.05; Student’s t-test).

Cancer metastasis is a complex and multistep process, requiring cancer cells to escape the confinement of the primary tumour, survive in the blood/lymph system and then to establish a new niche at a distant site. We previously showed that Kindlin-2 deficiency inhibited the growth of primary BC tumours using either human MDA-MB-231 or murine 4T1 BC cells lacking Kindlin-2 injected in mammary fat pads of mice^13^. The effect of Kindlin-2 on metastasis was, however, not reported. Therefore, we sought to investigate how the loss of Kindlin-2 influences BC metastasis. After the primary tumours were excised from the mammary sites, the mice were kept alive and monitored for an additional 3 weeks, after which mice were sacrificed, the lungs removed and metastasis nodules counted. In the MDA-MB-231 BC model, we found the number of lung nodules, which represent the metastasis foci, was reduced by more than 10-fold (p < 0.01) in mice implanted with the Kindlin-2-deficient (Kindlin-2 CRISPR) cells compared to their control (Scram CRISPR) counterparts (Fig. 3A,B). These results were replicated in murine 4T1 BC model, where CRISPR/Cas9-mediated knockout of Kindlin-2 (Fig. 3C), also resulted in ~10-fold decrease (p < 0.01) in the number of lung metastasis foci in mice implanted with the Kindin-2-deficient 4T1 cells, compared to the control cells (Fig. 3D,E).

**Figure 4:**
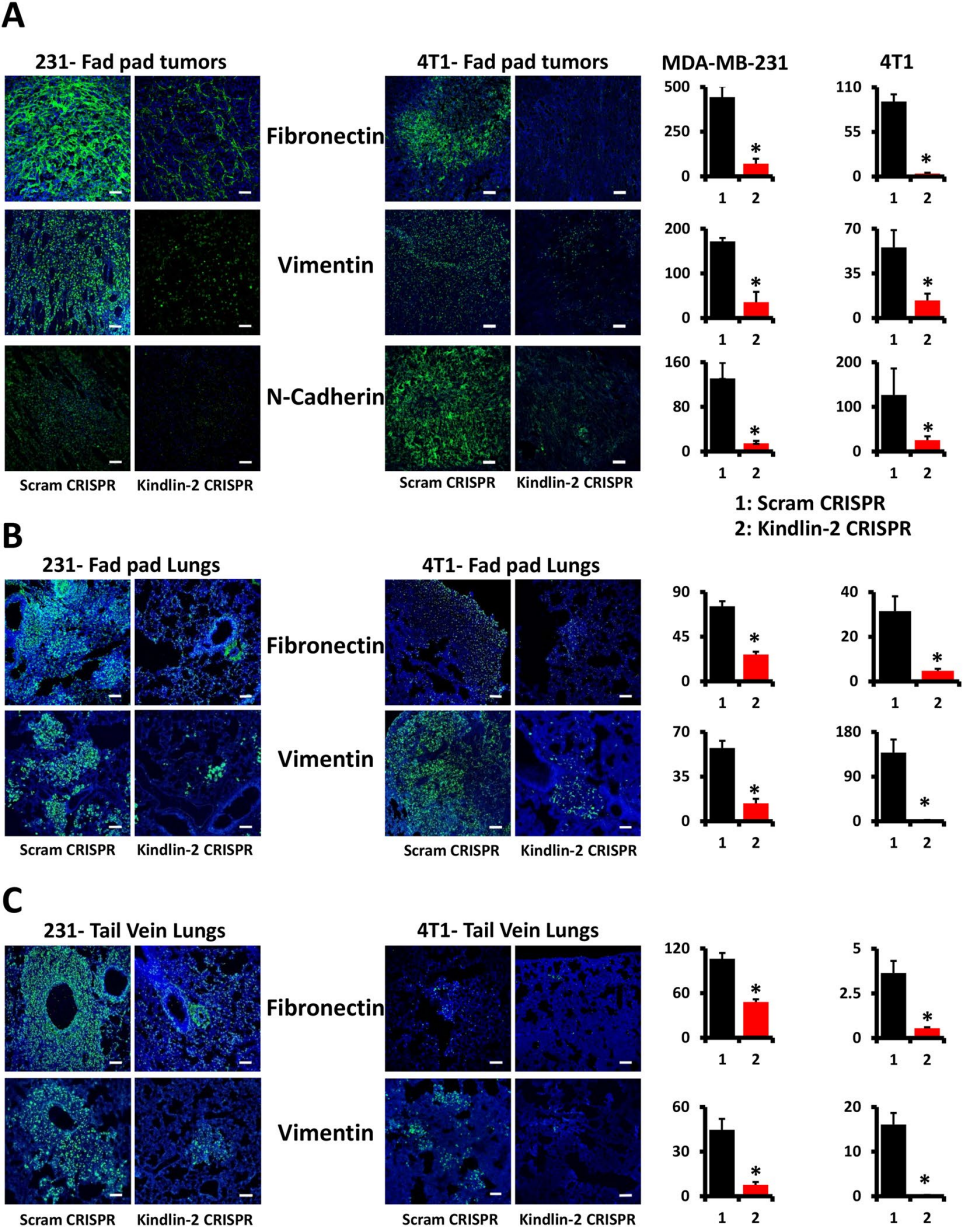
Loss of Kindlin-2 inhibits expression of key markers of the epithelial-to-mesenchymal transition program in BC tumours and metastases. (**A**) Representative images of MDA-MB-231- and 4T1-mammary fat pad-derived tumour sections stained in green for Fibronectin (top panels), Vimentin (middle panels) and N-Cadherin (bottom panels). Size bar, 146 µm. Quantification of staining is also shown. Data are expressed as mean ± SEM. *p < 0.001, n = 5 mice. (**B**) Representative images of lung sections from MDA-MB-231- and 4T1-mammary fat pad-implanted mice stained in green for Fibronectin (top panels) and Vimentin (bottom panels). Size bar, 146 µm. Quantification of staining is also shown. Data are expressed as mean ± SEM. *p < 0.001, n = 5 mice. (**C**) Representative images of lung sections from MDA-MB-231- and 4T1-tail vein-injected mice stained in green for Fibronectin (top panels) and Vimentin (bottom panels). Size bar, 146 µm. Quantification of staining is also shown. Data are expressed as mean ± SEM. *p < 0.001, n = 5 mice.

We also used the lung colonization assay, in which cancer cells were injected directly in the blood via the tail vein, to determine the effect of loss of Kindlin-2 on metastasis. Here again we found the lung metastasis nodules were reduced by ~10-fold (p < 0.01) when Kindlin-2-deficient MDA-MB-231 (Fig. 3F–H) or Kindlin-2-deficient 4T1 (Figs 3I and 4L) were injected into mice compared to their control counterparts. These data show that Kindlin-2 plays a critical role in the invasion and metastasis of BC tumours as demonstrated here with two different BC cells and in both the spontaneous and experimental metastasis assays.

### Loss of Kindlin-2 inhibits expression of key markers of the epithelial-to-mesenchymal transition program in BC tumours and metastases

EMT confers migratory and invasive capabilities, as well as metastatic capacities to cancer cells^3,4,7,8^. We therefore sought to determine whether the inhibition of BC metastasis that we found to be caused by loss of Kindlin-2 is a consequence of Kindlin-2 mediated regulation of EMT in the primary tumours. Among the several hallmarks of EMT are changes in expression levels of Fibronectin, Vimentin and N-Cadherin^15,16^. Immunostaining of mammary fat pad-derived tumour sections revealed a significant loss (p < 0.01) of all three EMT markers (Fig. 4A) in tumours derived from the Kindlin-2-deficient MDA-MB-231 or 4T1 (Kindlin-2-CRISPR) BC cells compared to their control counterparts (Scram CRISPR). We extended our immunostaining analyses to the lungs of mice implanted in the mammary fat pad with the Kindlin-2-deficient BC cells or their control derivatives. Here also, we found a significant (p < 0.01) loss of Fibronectin and Vimentin in the lungs of mice implanted with the Kindlin-2-deficient MDA-MB-231 or 4T1 BC cells compared to their counterpart controls (Fig. 4B). Similar results were found when we stained sections of lungs from mice that were injected via tail vein with the Kindlin-2-deficient MDA-MB-231 or 4T1 BC cells compared to their controls (Fig. 4C).

### Kindlin-2 is a direct target of microRNA miR-200b

**Figure 5:**
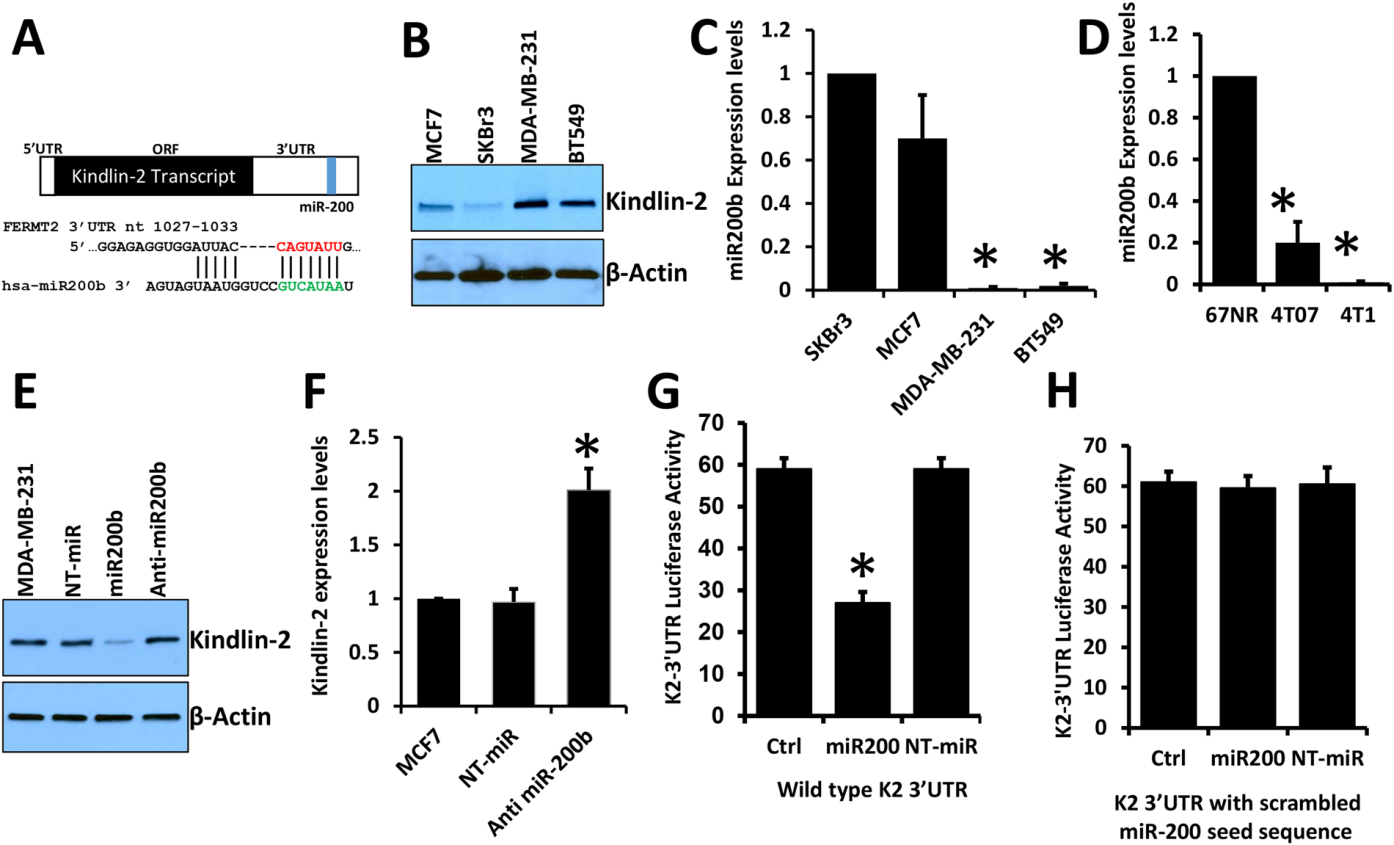
Kindlin-2 is a direct target of microRNA miR-200b. (**A**) Schematic representation FERMT2 (Kindlin-2) transcript showing the location of the seed sequence of microRNAs miR-200b within the 3′-UTR. The nucleotide sequence and location of the seed sequence with respect to the 3′UTR is shown, as well as its alignment with the miR-200b sequence. (**B**) Western blots of cell lysates from human BC cell lines with anti-Kindlin-2 antibody. β-Actin serves as a loading control. (**C**) Quantification of miR-200b levels in the human BC cell lines using qt-RT-PCR. Values were normalized to SKBr3 cells. (**D**) Quantification of miR-200b levels in the murine 4T1 BC series using qt-RT-PCR. Values were normalized to 67NR cells. (**E**) Western blots of cell lysates from MDA-MB-231 cells with the indicated transfections with anti-Kindlin-2 antibody. β-Actin serves as a loading control. (**F**) Quantification of Kindlin-2 transcript levels in MCF7 cells with the indicated transfections. Values were normalized to the untransfected cells. (**G**) Firefly luciferase reporter plasmid pmirGlo containing the wild-type 3′-UTR of Kindlin-2 was transiently transfected into MDA-MB-231 cells and treated as indicated. Luciferase activities were measured after 48 h and plotted after being normalized Renilla luciferase. (**H**) The same experiment was performed as in **G**, with the K2-3′UTR where the seed sequence of miR-200b was scrambled. Data are representative of 3 independent experiments (*p < 0.05; Student’s t-test).

The role of microRNAs in the regulation of the EMT program is well documented in the literature^17–19^. To further understand the underlying molecular and genetic or epigenetic mechanisms involved in the Kindlin-2-mediated promotion of BC metastasis through the activation of EMT, we investigated the role of microRNAs in the regulation of Kindlin-2 expression. *In silico* analyses, using several microRNA prediction algorithms (Target Scan, miRanda and PITA), identified microRNA miR-200b as one of the top predicted microRNAs that targets Kindlin-2 (Fig. 5A). In fact we showed that the seed sequence of miR-200b is a perfect match to a target sequence in the 3′UTR of Kindlin-2 (Fig. 5A). Further reinforcing our focus on miR-200b as a potential regulator of Kindlin-2 in BC are published findings that this microRNA is a master regulator of EMT in several cancer types^14,19–21^. First, we quantified the expression levels of Kindlin-2 and miR-200b in representative BC cell lines, and found Kindlin-2 expression levels to be low in the non-aggressive MCF7 and SKBR3 cells while it was comparatively high in the more aggressive and metastatic BT549 and MDA-MB-231 cells (Fig. 5B). This observation confirmed our previously published findings^13^. Of note, expression levels of miR-200b in these cell lines showed an inverse pattern: High in MCF7 and SKBR3, and low in BT549 and MDA-MB-231 cells (Fig. 5C). In fact, miR-200b was almost undetectable in these aggressive BC cell lines, which are of mesenchymal phenotype^22,23^, while it was abundant in the MCF7 and SKBR3 which are of epithelial phenotype (Fig. 5C)^22,23^. Also in the murine BC series in which Kindlin-2 expression levels were correlated positively with aggressiveness of these cells (low in the non-tumorigenic 67NR cells and the non-invasive 4T07 cells, and high in the aggressive and highly metastatic 4T1 cells^24^. miR-200b showed a negative correlation: its expression levels were more that 5-fold lower in the 4T07 cells compared to 67NR and was almost undetectable in the 4T1 cells. (Fig. 5D). Thus, we demonstrate an inverse correlation between Kindlin-2 and miR-200b in the aggressive and mesenchymal BC cells vs their non-aggressive and epithelial counterparts, which strongly suggest the involvement of Kindlin-2 in the miR-200b-mediated regulation of the EMT and that miR-200b is involved in the regulation of Kindlin-2 during this process.

Next, using both western blot (Fig. 5E) and qRT-PCR (Fig. 5F) analyses, we showed that transient transfection of MDA-MB-231 cells with miR-200b resulted in ~60% knockdown (p < 0.05) of Kindlin-2 protein levels (Fig. 5E), compared to the parental cells or those transfected with non-targeting microRNA (NT-miR). On the other hand, anti-miR-200b had no significant effect on Kindlin-2 protein levels (Fig. 5E). Conversely, when we transfected MCF7, a cell line that expresses high levels of miR-200b, with anti-miR-200b oligonucleotides (Fig. 5F), we observed a ~2-fold increase (p < 0.05) in Kindlin-2 transcript levels compared to the parental MCF7 cells or MCF7 cells transfected with the non-targeting microRNA (NT-miR). Thus, inhibition of Kindlin-2 expression is induced by miR-200b. To verify that miR-200b represses expression of Kindlin-2 by binding directly to Kindlin-2 transcripts, we used a luciferase gene reporter assay (pmirGlo^25^). MDA-MB-231 cells, which express very low levels of miR-200b, were engineered to express a pmirGlo construct where the wild-type form of Kindlin-2 3′-UTR (Wild-type K2-3′UTR) was subcloned upstream of the luciferase gene (231-wild-type K2-3′UTR). These cells remained either untreated (Ctrl) or transfected with either a non-targeting micro-RNA (NT-miR) or the miR-200b mimics (miR200). As expected, no difference in luciferase activity was found between the untreated 231-wild-type K2-3′UTR cells (Ctrl) and those same cells transfected with the non-targeting microRNA (NT-miR; Fig. 5G). In contrast, transfection of the 231-wild-type K2-3′UTR cells with miR-200b mimics (miR200) resulted in ~60% reduction (p < 0.05) in luciferase activity (Fig. 5G). Mutation of the seed sequence of miR-200b in the 3′-UTR of K2 (K2 3′UTR with scrambled miR-200 seed sequence) abrogated the effect of the exogenous miR-200b (Fig. 5H); the levels of luciferase activity did not show any significant difference when compared to the control cells or those treated with the non-targeting microRNA (Fig. 5H). These findings were replicated in the BT549 cells, confirming the generality of this observation. Thus, miR-200b specifically binds to its target sequence within the 3′UTR of Kindlin-2 and inhibits its expression.

### The miR-200b-mediated downregulation of Kindlin-2 inhibits BC metastasis

**Figure 6:**
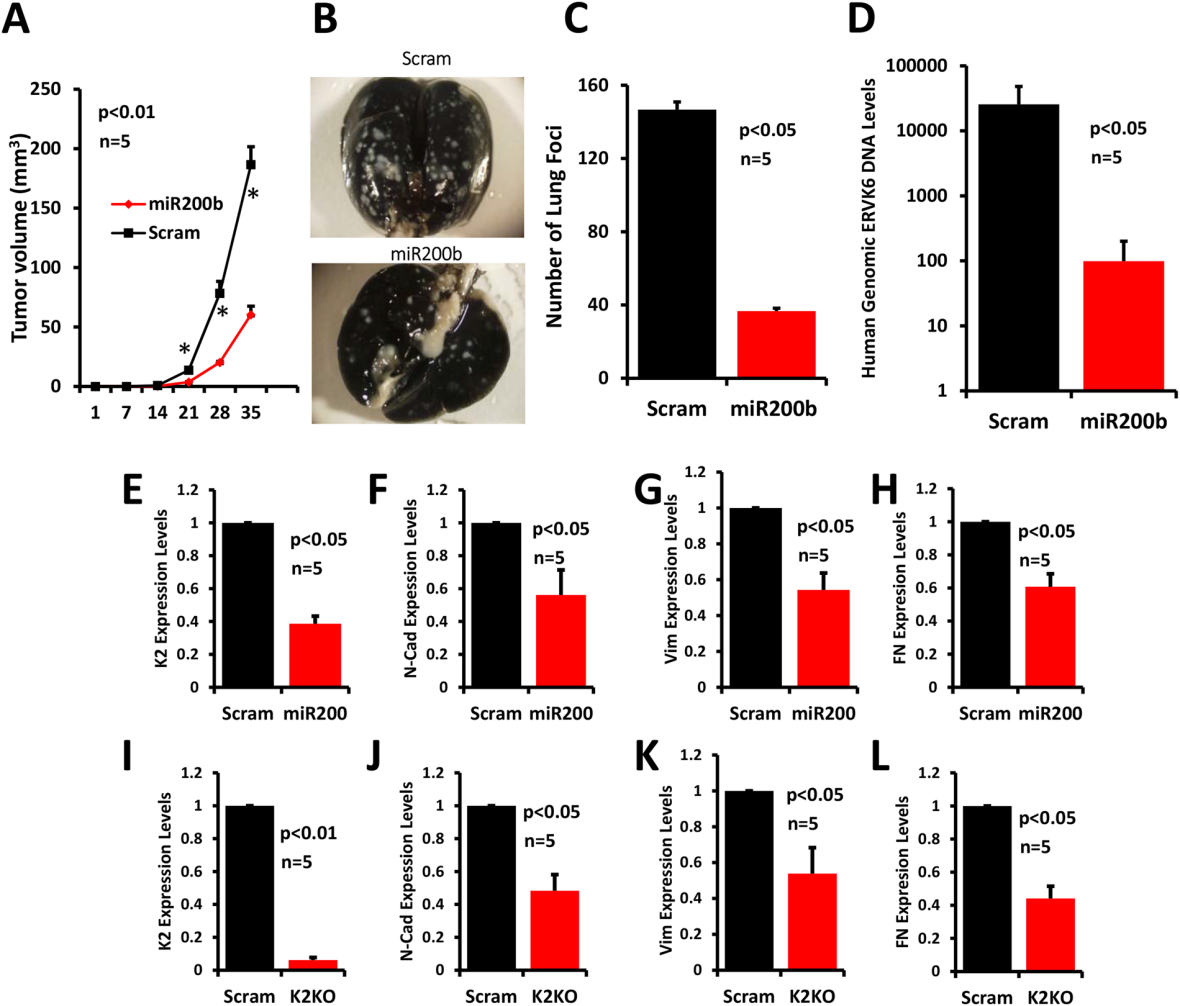
The miR-200b-mediated downregulation of Kindlin-2 inhibits BC metastasis. (**A**) Quantification of volume of tumours derived from implantation of Scram or miR-200b-expressing MDA-MB-231 cells into the mammary fat pads of NSG mice. (**B**) Representative images of black ink-filled lungs of NSG mice implanted into the mammary fat pads with Scram or miR-200b-expressing MDA-MB-231 cells. White spots represent metastasis foci. (**C**) Quantification of metastasis foci in the lungs shown in B. (**D**) Quantification of human genomic DNA in the lungs shown in B. Values were normalized to GAPDH. Data are expressed as mean ± SEM. *p < 0.001, n = 5 mice. (**E**–**L**) Quantification of Kindlin-2 (**E** and **I**), N-Cadherin (**F** and **J**), Vimentin (**G** and **K**) and Fibronectin (**H** and **L**) transcripts form lungs of NSG mice implanted into the mammary fat pads with Scram or miR-200b-expressing (miR200) MDA-MB-231 cells (**E**–**H**); or NSG mice implanted into the mammary fat pads with Scram or Kindlin-2-deficient (K2KO) MDA-MB-231 cells. Values were normalized to Scram. Data are representative of 3 independent experiments; *p < 0.05; Student’s t-test; n = 5 mice.

Having demonstrated that miR-200b specifically targets and inhibits Kindlin-2 expression *in vitro*, we extended our investigation to *in vivo* setting in the spontaneous metastasis assay. MDA-MB-231 cells with stable expression of miR-200b mimics or a scrambled control were injected into the mammary fat pads NSG mice and the metastasis burden was assessed 3 weeks after resection of the primary tumours by quantifying the number of lung metastasis foci. Overexpression of miR-200b slowed the growth of primary tumours; at the 5-week time point when all the primary tumours were removed, the average volume of the tumours derived from the miR-200-overexpressing MDA-MB-231 cells (miR200b) was more than 3-fold less (p < 0.01) than those derived from the control (Scram) cells (Fig. 6A). More importantly the number of lung metastasis foci was reduced by ~4-fold (p < 0.01) in the mice injected with the miR-200b-overexpressing MDA-MB-231 cells (miR200b), compared to their control (Scram) counterparts (Fig. 6B,C). We also used genomic DNA PCR to specifically amplify the human genomic DNA in the lungs of the mice injected with the modified human MDA-MB-1231 cells. We found the amounts of human DNA present in the lungs of mice injected with the miR-200b-overexpressin MDA-MB-231 (miR200b) to be more that 120-fold less than in the control (Scram) counterparts (Fig. 6D), confirming the inhibitory effect of miR-200b on metastasis. qt-RT-PCR analyses of total RNA from the primary tumours showed that Kindlin-2 mRNA levels were significantly lower in the tumours derived from the miR-200b-expressing MDA-MB-231 (miR200) cells (Fig. 6E) compared to those derived from the control (Scram) counterparts. The level of Kindlin-2 downregulation was comparable to that observed in the tumours derived from the Kindlin-2-deficient MDA-MB-231 (K2-KO) cells (Fig. 6I). Finally, we assessed the effect of overexpression of miR-200b on expression of EMT markers in the tumours using qt-RT-PCR. Expression levels of N-Cadherin, Fibronectin and Vimentin were significantly downregulated (p < 0.05) in the tumours derived from the miR-200b-overexressing MDA-MB-231 (miR200) cells compared to the tumours from the control (Scram) counterparts (Fig. 6F–H, respectively). The levels of downregulation of these EMT markers were similar to those observed in tumours from the Kindlin-2-deficient (K2KO) MDA-MB-231 cells (Fig. 6J–L). Together, these data demonstrate that Kindlin-2 modulates the growth and metastasis of BC tumours through the regulation of the EMT program and this regulation takes place downstream of miR-200b microRNA.

## Discussion

Aberrant activation of the EMT program endows tumours with migratory and invasive properties, as well as metastatic capabilities. Further, the EMT program also confers cancer cells with stem cell properties allowing the tumour cells to survive in circulation and establish distant colonies that often acquire resistance to therapeutic agents. EMT has been demonstrated as an essential early step during the invasion–metastasis cascade, whereby loss of E-Cadherin in cell junctions allows the dissolution of cell-cell contacts in primary tumours, thus enabling the release of invasive cells^4,7,19,26,27^. Concomitant with EMT, an increase in motility, migratory and invasive properties and in expression levels of EMT markers such as N-Cadherin, Fibronectin and Vimentin, take place which results in the increase in metastasis potential of cancer cells. This study demonstrates that Kindlin-2 supports BC metastasis and does so at least in part by regulating the EMT program downstream of microRNA miR-200b. We used several biochemical and cell imaging analyses, in combination with genetic manipulations, as wells as *in vitro* and *in vivo* mouse models, to investigate the role Kindlin-2 in BC metastasis, by regulating the expression of key EMT markers. We showed that (*i*) Kindlin-2 enhances tumour cell adhesion and spreading, as well as formation of focal adhesion complexes; (*ii*) Kindlin-2 activates the formation of invadopodia structures that are required for the degradation of the ECM; (*iii*) in *in vivo* mouse models, Kindlin-2 is required for the spreading and metastasis of BC tumours; (*iv*) the extent of tumour metastasis depends on the activation of the EMT program as measured by the expression of EMT markers; and (*v*) the Kindlin-2-mediated regulation of the EMT program depends on the capacity of miR-200b to regulate the expression levels of Kindlin-2 in tumour cells. Thus, we have established a miR-200b ⇔ Kindlin-2 signalling axis as key regulator of the EMT program, which in turn modulates BC tumours progression and metastasis.

Interest in Kindlin-2 was stimulated by the identification of its integrin co-activator function of integrins by binding to the cytoplasmic tail of beta-integrin, along with talin, to enhance affinity/avidity for ligands^28^. It is well-established that integrin are key players in the pathology of several cancers (reviewed in^11^); and the Kindlin-2-mediated co-activation of integrins was shown to regulate the function of integrins in cancers^29,30^. In fact, Kindlin-2 was found to be highly expressed in tumours from different types of cancers, including BC (reviewed in^11^). Our recently published study identified a new Kindlin-2/TGFβ/CSF-1 signalling axis that BC cells utilize to recruit and polarize host macrophage to drive tumour progression^13^. Whether the same signalling axis was sufficient to activate BC metastasis was not clear. The present study demonstrated that Kindlin-2 function is involved in the enhancement of BC metastasis by regulating the EMT program downstream of microRNA miR-200b. The miR-200 family is no stranger to EMT. In fact, the miR-200 microRNAs have been established as master regulators of the EMT program in both physiological and pathological conditions^19,21,27,31^. miR-200b was recently shown to suppress invasiveness and to modulate the cytoskeletal and adhesive machinery by targeting Kindlin-2 in oesophageal squamous cell carcinoma cells^32^. Our study revealed similar activity of miR-200b in BC by targeting Kindlin-2 *in vitro*; loss of adhesion and spreading, inhibition of focal adhesion formation, and inhibition of invadopodia and extracellular degradation. In addition, targeting and inhibition of Kindlin-2 by miR-200b in BC also resulted in the regulation of the EMT program leading to the inhibition of BC invasion and metastasis *in vivo*. Kindlin-2 was also reported to promote BC invasion through epigenetic silencing of members of the miR-200 gene family^33^. Therefore, our study together with the data published by Yu *et al*.^33^ show that EMT and the resultant invasion metastasis cascade in BC seems to be regulated trough a miR-200b/Kindlin-2 feedback loop.

The miR-200b/Kindlin-2/EMT signalling axis may not be the only mechanisms through which Kindlin-2 regulates cancer metastasis. Recent studies have involved Kindlin-2 in other oncogenic signalling pathways responsible for the activation of cancer metastasis, including a recent study by Lin *et al*.^34^, that showed Kindlin-2 promotes metastasis of hepatocellular carcinoma through Wnt/β-catenin signalling. It is possible that both signalling mechanisms may contribute together to the Kindlin-2-mediated regulation of BC metastasis, as the Wnt/β-catenin signalling has been reported to be regulated by Kindlin-2 in BC^35^.

The finding from this study on the role of Kindlin-2 in the regulation of the metastasis phenotype is a continued effort that was built upon our recently published data that demonstrated a key role for Kindlin-2 in the modulation of the tumour microenvironment to support BC growth and progression^13^, as well as its involvement in the molecular mechanisms of tumour resistance to therapy^36^. Together these studies support the function of Kindlin-2 as a major regulator of several key hallmarks of cancer^37,38^; and make a strong argument for the development of therapeutic strategies to target Kindlin-2 in BC as well as other cancers.

## Materials and Methods

### Ethics statement

All animal studies were performed under protocols approved by the Institutional Animal Care and Use Committee at Cleveland Clinic. This study used 6- to 8-week-old female NSG or BALB/C mice (Jackson Laboratory, Farmington, CT). All methods were performed in accordance with the guidelines and regulations set and approved by the Cleveland Clinic.

### Cell Lines and Reagents

BC cell lines were obtained from American Type Culture Collection (ATCC), and maintained according the manufacturer’s protocols. Cell lines were also routinely authenticated by STR DNA fingerprinting analysis. 4T1 cells were engineered to stably express firefly luciferase by transfection with pNifty-CMV-luciferase and selection (500 μg/ml) with Zeocin (Invitrogen, Carlsbad, CA). Kindlin-2-KO cells were generated by lentiviral transduction. We used two independent and verified Kindlin-2-specific sgRNAs for human and mouse Kindlin-2 and a scrambled sgRNA (i.e., nonsilencing sgRNA). Loss of Kindlin-2 expression was determined by Western blot.

### Western blot analyses

Cells were grown in 6-well tissue culture plates until confluency reached ~80%. Cells were then washed twice with ice-cold PBS and lysed on ice with RIPA buffer. The crude lysates were transferred to prechilled tubes and centrifuged at 13K RPM for 15 min at 4 °C. Cleared cell lysates were denatured with SDS sample buffer and resolved (~25 ug) on a 4–20 gradient SDS acrylamide gels (BioRad), followed by transfer to polyvinylidene difluoride membranes (BioRad). Membranes were incubated in 5% bovine serum albumin in PBST buffer for 1 h at room temperature, followed by incubation with the primary antibody (as specified) overnight at 4 °C. Membranes were then washed 3 times with PBST buffer and incubated in the appropriate secondary antibody at room temperature for 1 h, and the signals were developed using the Western Lights chemiluminescence detection kit (PerkinElmer Life Sciences).

### Cell spreading and adhesion assays

Cell adhesion assays were carried out by seeding cells on round coverslips that were pre-coated for 1 hour with 10 μg/mL collagen I (Sigma) in 12-well plates. Cells were allowed to adhere for 1 h or spread overnight in a 37 °C CO2 incubator. Spread cells were washed twice with PBS then fixed with 4% Paraformaldehyde for 20 minutes and washed twice with PBS. Fixed cells were permeabilized with 0.1% Triton X-100 in PBS for 15 min, washed with PBS, then stained for 1 hour with Alexa Fluor 568-conjugated phalloidin (Invitrogen, Molecular Probes). After washing, the coverslips were on slides on a drop of DAPI-containing immune-fluor mounting medium (MP Biomedicals). Images were acquired with a fluorescent microscope (Leica) equipped with digital camera using Image-Pro Plus version 5.1.2.59. Cell adhesion was quantified by counting the number of cells (nuclei) and averaged to 20 fields, while cell spreading was quantified with ImageJ software version 1.43 u and the results were plotted as the total surface area covered by cells, in square micro meter, averaged to 300 cells.

### Invadopodia formation and extracellular matrix degradation Assays

FITC-gelatine degradation assays were performed according to the procedures described in our previously published study^39^, following the manufacturer’s instructions (Invitrogen, CA). We used fluorescence confocal microscopy to document invadopodia and ECM degradation, as described^40,41^. The ImageJ software was used to analyse the data and for 3D reconstruction of images.

### Primary Tumour Growth, Bioluminescence Imaging and Metastasis assays

Parental (scram) or Kindlin-2-deficient MDA-MB-231 cells (106 cells per mouse, n = 5) were implanted into the mammary fat pads of female NSG mice. Tumour growth was followed by twice weekly monitoring of tumour volume with digital Vernier callipers. Luciferase-expressing parental (scram) or Kindlin-2-deficient 4T1 cells (10,000 cells per mouse, n = 5) were implanted into the mammary fat pads of female BALB/c mice. Tumour growth was quantified using bioluminescence imaging as described^13^. In the metastasis, cells (2 × 10^6^) suspended in 0.15 ml of sterile PBS were injected using a 28-gauge needle into a tail vein of 6- to 8-week-old female NSG mice. Mice were sacrificed 5 weeks later and the recovered lungs were filled and stained with 15% black India ink using a 19-gauge needle, administered through the trachea. Tumour cells appear as white spots on the lung since they do not take up the ink. Metastasis foci (white spots) were counted and the results were plotted as average number of foci per lobe.

### Immunohistochemistry

Tumour and lung tissues were harvested and snap-frozen in optimal cutting temperature (OCT) medium (Sakura Finetek, Torrance, CA, USA), and 8-μm sections were prepared. Tumour sections were stained with the following antibodies: rabbit anti-fibronectin (Millipore, Temecula, CA, USA), rabbit anti-vimentin (Cell Signaling Technology, Danvers, MA, USA), rabbit anti-N-cadherin (Thermo Scientific, Rockford, IL, USA), followed by Alexa 488 or Alexa 568-conjugated goat anti-rabbit IgG. Lung sections were stained with hematoxylin (Vector Laboratories, Burlingame, CA, USA) and eosin (Fisher Scientific Co., Kalamazoo, MI, USA) following the manufacturer’s protocol. We used fluorescent or bright-field imaging microscopy (Leica, Wetzlar, Germany) and ImagePro Plus Capture and Analysis software (Media Cybernetics, Rockville, MD, USA) to analyse the stained sections. We used Image Pro-Plus software to quantify stained areas from 10–15 independent fields/sections.

### Genomic DNA Assay

The human genomic PCR was performed as previously described^42^. The human-specific ERVK6A gene can be identified in human DNA (MDA-MB-231) but not in mouse DNA (mouse lung). The presence of the human DNA sequences in the mouse lungs is an indication of the presence of human cells in these tissues. ERVK6A was amplified form DNA of lungs derived from control mice (scram 231 cells) or mice injected with 23-K2-KO cells. The mouse Wave3 gene is used as a control for integrity of the mouse DNA in each sample.

### Site-directed Mutagenesis, and Luciferase promoter assay

The 3′-UTR of Kindlin-2 was PCR-amplified using K2 cDNA as a template and subcloned into pmirGlo vector downstream of the firefly luciferase expression cassette (Promega, Madison, WI). The correct sequence and orientation were verified by sequencing. We used the QuikChange site-directed mutagenesis kit (Stratagene, La Jolla, CA), as per the manufacturer’s instructions, to generate the K2 3′-UTR variants, where seed sequences that are recognized by miR200 microRNA were deleted. pmirGlo reporter plasmids (1 μg total plasmid amount) were transfected with Lipofectamine 2000 (Invitrogen) into the specified cancer cells, seeded in 12-well plates (3 × 104 cells/well). Cells were collected after 48 h for assay using the dual-luciferase reporter assay system (Promega). For co-transfection experiments, microRNA mimics or miR negative control (5 nM) or anti-miR200b microRNA inhibitor or anti-miR negative control (20 nM) were added to the transfection mix.

### Antibodies and reagents

Mouse monoclonal anti-K2, clone 3A3 (1:2000, EMD Millipore, Bedford, MA); goat horseradish peroxidase-conjugated anti-mouse IgG (1:2,000) and goat horseradish peroxidase-conjugated anti-rabbit IgG (1:2,000) were from Calbiochem. Vecta-shield with 4′,6-diamidino-2-phenylindole was from Vector Laboratories. Gel electrophoresis reagents were from BioRad.

### Real-time quantitative RT-PCR

We used TRIzol reagent (Invitrogen, Camarillo, CA) to extract total RNA from cancer cell lines and tumour tissues using, according to the manufacturer’s instructions. Quantitative real-time PCR was performed as described previously^36,41,43^. Oligonucleotide primers used) for qt-RT-PCR (Supplementary Table 1) were obtained from Qiagen (Germantown, MD).

### Immunostaining

Tissues were collected at the designated times and snap-frozen in optimal cutting temperature (OCT) medium (Sakura Finetek, Japan), and 8-μm sections were prepared and stained with the following antibodies; Rabbit anti-fibronectin (1:100) (Millipore, Temecula, CA, USA), rabbit anti-vimentin (1:100) (Cell Signalling Technology, Danvers, MA, USA), rabbit anti-N-cadherin (1:1000) (Thermo Scientific, Rockford, IL, USA), as described previously^13,41^. Stained sections were analysed using fluorescent or bright-field imaging microscopy (Leica, Germany) and ImagePro Plus Capture and Analysis software (Media Cybernetics, Rockville, MD). Vimentin, Fibronectin and N-Cadherin positive areas were quantified in 15 independent fields/section using Image Pro-Plus software^41,44^.

### Statistical analysis

Experiments were done in triplicate and analysed using the Student’s t-test. In calculating two-tailed significance levels for equality of means, equal variances were assumed for the two populations. Results were considered significant at p < 0.05.

### Data availability

The datasets generated and analysed during this study that are not included in the published article are available from the corresponding author upon reasonable request.

## Electronic supplementary material

Supplementary Information
