# Supplementary material for: The Kindlin-2 regulation of epithelial-to-mesenchymal transition in breast cancer metastasis is mediated through miR-200b
Source: Sci Rep. 2018 May 9;8:7360. doi: 10.1038/s41598-018-25373-0 (PMC5943603; doi:10.1038/s41598-018-25373-0)
Supplement: Supplementary file 1 — Supplementary Information [file 41598_2018_25373_MOESM1_ESM.pdf]

Supplementary Information

The Kindlin-2 regulation of epithelial–to-mesenchymal transition in breast cancer metastasis is mediated through miR-200b

Khalid Sossey-Alaoui[1]*, Elzbieta Pluskota[1], Dorota Szpak[1], William P Schiemann[2], Edward F. Plow[1].

Supplementary Table 1: PCR Primers Information:

Oligonucleotide Sequence of sgRNAs used for CRISPR/Cas9 gene knockdown

Human Kindlin-2-sgRNA-1	5'-AGGCGTGATGCTTAAGCTGG-3’
Human Kinlin-2-sgRNA-2	5’-GGTATACTTGCTGTCAGTCA-3’
Mouse Kindlin-2-sgRNA-1	5'-CGGGGAGGTGCACATCGGAG-3'
Mouse Kindlin-2-sgRNA-2	5'-GTGACCGGGGAGGTGCACAT-3'

Primers for SYBER Green real-time quantitative PCR

Human Kindlin-2	Qiagen Catalog Number PPH15416B
Mouse Kindlin-2	Qiagen Catalog Number PPM33282A
Human Fibronectin	Qiagen Catalog Number PPH00143B
Mouse Fibronectin	Qiagen Catalog Number PPM03786A
Human Vimentin	Qiagen Catalog Number PPH00417F
Mouse Vimentin	Qiagen Catalog Number PPM04780B
Human N-Cadherin	Qiagen Catalog Number PPH00636F
Mouse N-Cadherin	Qiagen Catalog Number PPM03840C
Human MMP2	Qiagen Catalog Number PPH00151B
Human MMP9	Qiagen Catalog Number PPH00152E
Human GAPDH	Qiagen Catalog Number PPH00150F
Mouse GAPDH	Qiagen Catalog Number PPM03661C
Human ERVK6	Qiagen Catalog Number PPH60565A

Primers for site directed Mutagenesis

Deleted-miR-200b-F	5'- AGGTGGATTACCATGTTCAAT-3'
Deleted-miR-200b-R	5'- ATTGAACATGGTAATCCACCT-3'

Sequencing primers for the pmirGlo vector

pmirGlo-F	5'-GAAGCTGAGTTGGCTGCT-3'
pmirGlo-R	5'-CACTGCATTCTAGTTGTGGT-3'

microRNA 200b

Mature sequence	UAAUACUGCCUGGUAAUGAUGA
miR-200b mimic	Ambion Product Number: AM17100; Product ID: PM10492
miR Negative Control	Ambion Product Number: AM17110
Anti-miR-200b Inhibitor	Ambion Product Number: AM17000; Product ID: AM10492
Anti-miR Negative Control	Ambion Product Number: AM17010

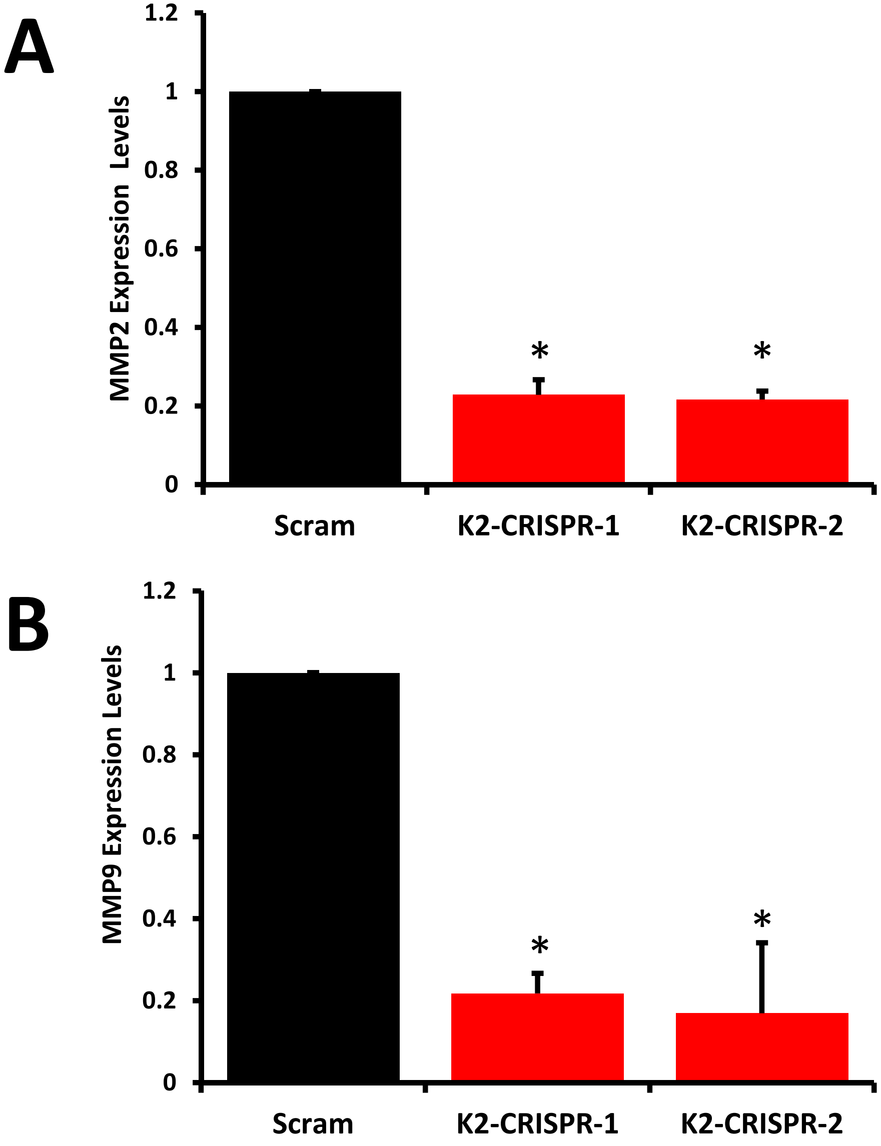

Figure S1: CRISPR/Cas9-mediated knockout of Kindlin-2 inhibits MMP2 and MMP9 expression.
Quantification of MMP2 (A) and MMP9 (B) levels in the control (Scram) and K2KO (K2-CRISPR) MDA-MB-231 BC cell lines using qt-RT-PCR. Values were normalized to the Scram cells. Data are representative of 3 independent experiments (*, $p < 0.05$; Student's t-test)
